# *Morinda citrifolia* (Noni) Fruit Juice Reduces Inflammatory Cytokines Expression and Contributes to the Maintenance of Intestinal Mucosal Integrity in DSS Experimental Colitis

**DOI:** 10.1155/2017/6567432

**Published:** 2017-01-17

**Authors:** Beatriz Coutinho de Sousa, Juliana Reis Machado, Marcos Vinicius da Silva, Thiago Alvares da Costa, Javier Emilio Lazo-Chica, Thatiane do Prado Degasperi, Virmondes Rodrigues Junior, Helioswilton Sales-Campos, Elizabeth Uber Bucek, Carlo José Freire Oliveira

**Affiliations:** ^1^Institute of Natural and Biological Sciences, Federal University of Triângulo Mineiro, 38025-180 Uberaba, MG, Brazil; ^2^Institute of Tropical Pathology and Public Health, Federal University of Goias, 74605-050 Goiania, GO, Brazil; ^3^Department of Pharmaceutical Sciences, University of Uberaba, 38050-501 Uberaba, MG, Brazil

## Abstract

*Morinda citrifolia* L. (noni) has been shown to treat different disorders. However, data concerning its role in the treatment of intestinal inflammation still require clarification. In the current study, we investigated the effects of noni fruit juice (NFJ) in the treatment of C57BL/6 mice, which were continuously exposed to dextran sulfate sodium (DSS) for 9 consecutive days. NFJ consumption had no impact on the reduction of the clinical signs of the disease or on weight loss. Nonetheless, when a dilution of 1 : 10 was used, the intestinal architecture of the mice was preserved, accompanied by a reduction in the inflammatory infiltrate. Regardless of the concentration of NFJ, a decrease in both the activity of myeloperoxidase and the key inflammatory cytokines, TNF-*α* and IFN-*γ*, was also observed in the intestine. Furthermore, when NFJ was diluted 1 : 10 and 1 : 100, a reduction in the production of nitric oxide and IL-17 was detected in gut homogenates. Overall, the treatment with NFJ was effective in different aspects associated with disease progression and worsening. These results may point to noni fruit as an important source of anti-inflammatory molecules with a great potential to inhibit the progression of inflammatory diseases, such as inflammatory bowel disease.

## 1. Introduction

The potential anti-inflammatory activities of several natural compounds in the downregulation of key players in the development of inflammation have been explored in different scenarios including the modulation of cytokines, transcription factors, enzymes, and the production of protein and nonprotein inflammatory mediators. These activities include the modulation of cytokines (e.g., IL-6, TNF-*α*, IFN-*γ*, IL-17, and IL-12), transcription factors, enzymes (e.g., myeloperoxidase-MPO and cyclooxygenase COX-1 and COX-2), and also the production of nitric oxide (NO) [1–3]. Due to their importance in controlling inflammation, therapies targeting such molecules have been suggested as possible aids in the prevention and/or treatment of inflammatory diseases, such as rheumatoid arthritis [4], dermatitis [5], and inflammatory bowel disease (IBD) [6]. IBDs are chronic inflammatory diseases of the gastrointestinal tract, which are clinically present as one of the two disorders, Crohn's disease (CD) or ulcerative colitis (UC) [7, 8]. These pathologies are of special interest since they affect millions of people worldwide, and current therapies are still not fully effective in controlling disease progression or preventing the occurrence of side effects [6].

*Morinda citrifolia* L. (noni) belongs to the Rubiaceae family, and it is a source of natural molecules that has been used as a medicinal plant by the Polynesians for more than 2,000 years [9]. So far, several bioactive compounds have been isolated from noni fruits, including fatty acids, flavonoids, polysaccharides, and sterols [10–13]. The anti-inflammatory potential of noni fruit compounds has been demonstrated in an experimental model of* Helicobacter pylori* infection in which ethanol and ethyl acetate extracts were used. These extracts were able to reduce both neutrophil chemotaxis and production of inducible nitric oxide (iNOS) and COX-2 [14]. Accordingly, C57BL/6 mice orally treated with noni fruit juice at 500 mg kg^−1^ day^−1^ for 60 days showed reduced inflammatory infiltrate and cytokine expression for IL-12, TNF-*α*, TGF-*β*, and IL-10, in the footpad infected with* Leishmania amazonensis* [15]. It is important to mention that cytokines such as IL-12, IL-6, TNF-*α*, IFN-*γ*, IL-17, and IL-23 are associated with the development and worsening of IBD, so they have been approached differently in order to treat this inflammatory disorder [7]. Furthermore, the anti-inflammatory potential of* Morinda citrifolia* leaf extract was shown by the reduction of TNF-*α*, IL-1*β*, and NO levels in macrophages after stimulation with lipopolysaccharide (LPS) [16]. Even though the role of noni fruit compounds in controlling inflammatory players is of special relevance, their effects in the development of intestinal inflammation are still poorly explored.

Therefore, this study showed the effects of noni fruit juice on cytokine interplay and intestinal architecture in a murine model of dextran sulfate sodium-induced colitis as underlying mechanisms for its immunomodulatory activity.

## 2. Materials and Methods

### 2.1. Collection and Botanical Identification of the Plant

The fruits used in this study were obtained from the monoculture of 150 noni plants on* Fazenda Boa Vontade*, a farm in the municipality of Araguari, Triângulo Mineiro/MG, Brazil, at coordinates 18°43′47.23′′S, 48°6′49.50′′O (data from Google Earth, 2013). All the specimens were prepared according to conventional herborization techniques [17] and deposited in the herbarium of the Federal University of Uberlândia (HUFU Herbarium) under the registration number HUFU-67210, as* Morinda citrifolia* L. (Rubiaceae).

### 2.2. Juice Extraction Process

*Morinda citrifolia* (noni) juice was prepared in the Laboratory of Pharmacognosy of University of Uberaba, in Uberaba, Minas Gerais, Brazil.* M. citrifolia* fruit was manually and randomly collected from 150 plants, washed in ozonated water, and kept at room temperature for 3–5 days. The fruits were mechanically depulped using a fruit depulper and, after seed removal, the resulting pulp was centrifuged at 4,000 rpm under refrigeration until the supernatants were clear, and it was then considered 100% (v/v) juice and stored at −70°C until further use.

### 2.3. Animal Studies

Male C57BL/6 mice aged 6–8 weeks and weighing 20–25 g were housed in specific pathogen-free and standard-controlled environmental conditions at constant temperature (25°C) on a 12-hour light/dark cycle, with* ad libitum* access to food and water, in the animal housing facility of the Federal University of Triângulo Mineiro (UFTM), Brazil. All animal studies were performed in accordance with the Institutional Animal Care and Use Committee of UFTM under protocol 275. The experiments were performed with 8 mice/groups, as follows:* saline*, healthy control mice treated with saline; DSS 2.5%, mice exposed to dextran sulfate sodium (DSS); DSS 2.5% +* pure noni*, mice exposed to DSS and treated with pure noni fruit juice; DSS 2.5% +* noni* 1 : 10, mice exposed to DSS and treated with a 1 : 10 dilution of noni fruit juice; and DSS 2.5% +* noni* 1 : 100, mice exposed to DSS and treated with a 1 : 100 dilution of noni fruit juice. A volume of 100 *μ*l per mouse was administered orally for 9 consecutive days.

### 2.4. DSS-Induced Colitis and Clinical Assessment

Colitis was induced by 2.5% DSS (MP Biomedicals, Illkirch, France, Molecular weight: 36,000–50,000 kDa) continuously added to the drinking water for 9 consecutive days for sample collection. In addition to recording daily food and water intake, body weight changes and clinical signs of disease were also assessed every day so as to obtain a clinical disease score for every mouse. Each sign presented by the animals corresponded to one point, and the sum of points for each mouse defined a clinical score. Clinical scores were determined as previously described herein [18].

### 2.5. Euthanasia and Sample Collection

The mice were euthanized on day 9, and the colon was removed for further analysis. The colon samples were divided into smaller sections that were immersed into PBS/10% formaldehyde for paraffin embedding or were immediately frozen in liquid nitrogen for quantification of myeloperoxidase (MPO) or nitric oxide (NO) activity by enzymatic assays. Moreover, one intestinal section from each mouse was collected in a solution containing protease inhibitors (Complete®, Roche Pharmaceuticals, Mannheim, Germany) for cytokine quantification by enzyme-linked immunosorbent assay (ELISA).

### 2.6. Myeloperoxidase (MPO) and Nitric Oxide (NO)

Briefly, for MPO assay, the sections were homogenized and erythrocytes were lysed. The pellet obtained after centrifugation was resuspended, followed by three freeze-and-thaw cycles. After centrifugation, the supernatant was placed in 96-well plates and revealed with tetramethylbenzidine (TMB) substrate (BD OptEIA™, San Diego, CA) at 37°C. The reaction was stopped and readings were performed in a spectrophotometer at 450 nm. MPO activity was determined as previously described [19]. Results were normalized to the dry weight of each intestinal section and expressed as optical density per gram of tissue (nm/g of tissue).

For NO measurement, the nitrite accumulated in intestinal homogenates was measured as an indicator of NO production using Griess reaction [20]. Then, 100 *μ*L of tissue homogenate was mixed with 100 *μ*L of Griess reagent, which is composed by equal volumes of 1% (w/v) sulfanilamide in 5% (v/v) phosphoric acid, and 0.1% (w/v) naphthyl-ethylenediamine-HCl, and incubated at room temperature for 10 min. The absorbance was measured at 540 nm in a 96-well plate reader (Perkin Elmer Cetus, CA, USA). The amount of nitrite in the samples was calculated using linear regression analysis of the absorbance of the serial dilution of sodium nitrite standard curve. Results were normalized to the dry weight of each intestinal section and expressed as picogram per milliliter per gram of tissue (pg/ml/g).

### 2.7. Cytokine Quantification by ELISA

Cytokines IL-10, IL-17, IFN-*γ*, TNF-*α*, IL-12, IL-4, and IL-23 were quantified in tissue homogenates by ELISA according to the manufacturer's instructions (BD Biosciences, San Jose, CA, USA). Results were normalized to the dry weight of each intestinal section and expressed as nanogram per milliliter per gram of tissue (ng/mL/g of tissue).

### 2.8. Histology and Histopathological Analysis

In order to assess the microscopic damage, the intestinal sections were cut longitudinally, washed with PBS, fixed in 10% buffered formalin for 24 h, and then processed for paraffin embedding followed by microtome sectioning. Tissue sections (5 *μ*m) were obtained and stained with hematoxylin and eosin (H&E). For histopathological analysis, the mucosa, submucosa, muscle layers, and serosa were evaluated. These intestinal sections were also assessed for the presence of edema, inflammatory infiltrate, and epithelial abnormalities.

Images were captured using a digital video camera (Evolution MP 5.0 color Media Cybernetics, Silver Spring, MD, USA), with a 10x objective, coupled to a light microscope (Nikon Eclipse 50i, Melville, NY, USA). Morphometry was performed using Image-Pro Insight (Media Cybernetics). The inflammatory infiltrate was measured based on the damaged area containing inflammatory infiltrate divided by the total area of tissue visualized in the acquired image and expressed as a percentage (%). A trained pathologist who was blinded to treatment performed the histopathological analysis.

### 2.9. Data Analysis and Statistics

Normal distribution and homogeneous variance were tested for all of the variables. When the distribution was considered normal and the variance was homogeneous, parametric tests were used: unpaired Student's *t*-test or one-way ANOVA followed by Tukey's post hoc test. In cases of non-Gaussian distribution of data, the following nonparametric tests were used: Mann–Whitney test or Kruskal-Wallis test accompanied by Dunn's post hoc test. The results were expressed as mean ± SD. The differences observed were considered significant when *p* < 0.05 (5%). Statistical analysis was performed using GraphPad Prism, version 5.0 (La Jolla, CA, USA).

## 3. Results

### 3.1. Treatment with Noni Fruit Juice and Disease Outcome

**Figure 1:**
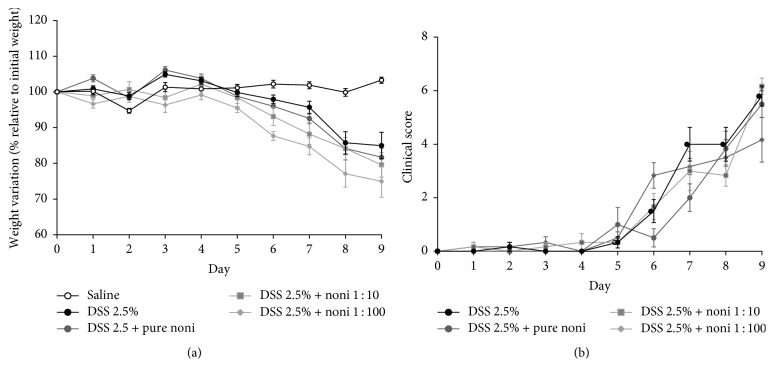
Treatment with noni fruit juice and disease outcome. C57BL/6 mice were exposed to dextran sulfate sodium (DSS) 2.5% and treated daily with noni fruit juice. On day 9, mice were euthanized to obtain intestinal sections. (a) Percentage of weight change. (b) Clinical disease score.* Saline*, healthy control mice treated with saline; DSS 2.5%, mice exposed to DSS; DSS 2.5% +* pure noni*, mice exposed to DSS and treated with the pure noni fruit juice; DSS 2.5% +* noni* 1 : 10, mice exposed to DSS and treated with a 1 : 10 dilution of the fruit juice; and DSS 2.5% +* noni* 1 : 100, mice exposed to DSS and treated with a 1 : 100 dilution of the fruit juice.

First, in order to assess whether noni fruit juice was able to prevent weight loss and the outcome of DSS-induced colitis, the mice were exposed to DSS for 9 days and then treated with the fruit juice, as described in* Materials and Methods*. The noni fruit juice group did not seem to reduce weight loss when compared to their control counterparts (DSS 2.5%) (Figure 1(a)). Furthermore, regardless of the concentration used, there was no effect on the presentation of clinical signs of disease in the mice treated with noni fruit juice in relation to the untreated mice (Figure 1(b)).

### 3.2. Noni Fruit Juice Consumption Inhibits Inflammation and Preserves Intestinal Architecture in a Dose-Dependent Manner

**Figure 2:**
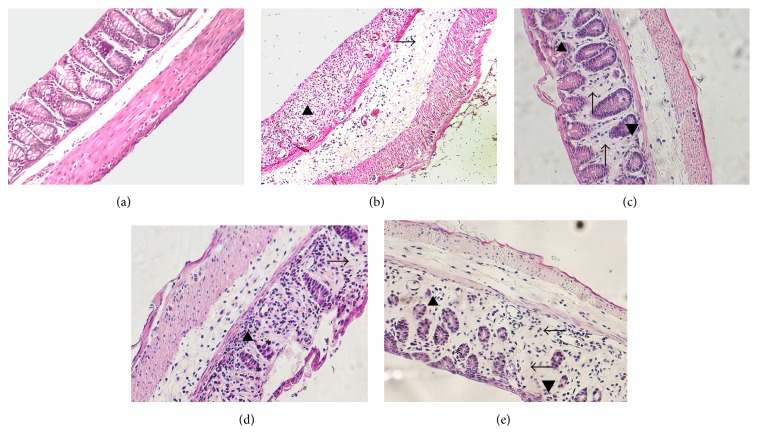
Noni fruit juice consumption preserves intestinal architecture in a dose-dependent manner. C57BL/6 mice were exposed to dextran sulfate sodium (DSS) 2.5% and treated daily with noni fruit juice. The colon was collected on day 9 for histopathological analysis. (a) Healthy mice without colitis; (b) mice exposed to DSS: lamina propria with moderate edema and mononuclear cell infiltration (arrowhead), submucosa with moderate mononuclear infiltrate and severe edema (arrow); (c) mice exposed to DSS and treated with pure noni fruit juice: lamina propria with moderate edema (arrow) and mononuclear infiltrate (arrowhead); (d) mice exposed to DSS and treated with noni fruit juice diluted 1 : 10: crypts with preserved irregularities (asterisk); lamina propria with moderate edema (arrow) and mononuclear infiltrate (arrowhead); (e) mice exposed to DSS and treated with noni fruit juice diluted 1 : 100: regular and atrophic crypts (asterisk); lamina propria with moderate edema (arrow) and mononuclear infiltrate (arrowhead).

After that, since no macroscopic effects were observed after treatment with noni fruit juice, we aimed to determine if the consumption of fruit juice had any microscopic effect on intestinal architecture. It was observed, in a dose-dependent manner, that the group treated with noni fruit juice was able to preserve their intestinal architecture. Healthy mice (Figure 2(a)) had the epithelium surface preserved, rectilinear crypts, composed by habitual number of goblet cells. In the lamina propria, the usual mononuclear infiltrate was visualized. The submucosa, muscular, and serous muscles had normal architecture. Mice exposed to DSS had erosions on the surface of intestinal epithelium and absence of crypts (Table 1). The lamina propria showed moderate edema and mononuclear cell infiltration (Figure 2(b), arrowhead), the submucosa had moderate mononuclear infiltrate and severe edema (Figure 2(b), arrow), and the muscular layer was slightly thickened, with colitis activity. In mice exposed to DSS and treated with pure noni juice, crypt irregularities were scarce and mild (Table 1), and the lamina propria had moderate edema (Figure 2(c), arrow) and mononuclear infiltrate (Figure 2(c), arrowhead); however, only a mild mononuclear infiltrate and moderate edema were detected in the submucosal layer, and the muscular layer was slightly thickened. On the other hand, in mice treated with noni fruit juice diluted 1 : 10, crypts had their irregularities preserved (Figure 2(d), asterisk), the lamina propria showed both moderate edema (Figure 2(d), arrow) and mononuclear infiltrate (Figure 2(d), arrowhead), and the submucosa only had a mild mononuclear infiltrate and moderate edema (Table 1). In the mice treated with noni fruit juice diluted 1 : 100, crypts were irregular and atrophic (Figure 2(e), asterisk), the lamina propria had moderate edema and mononuclear infiltrate, and the submucosal layer showed a mild mononuclear infiltrate and moderate edema (Table 1). In general, the architecture of intestinal crypts was preserved in the mice treated with noni fruit juice, and it was more preserved in mice that received noni juice at 1 : 10 and 1 : 100 dilutions in comparison with those treated with pure noni or DSS.

**Figure 3:**
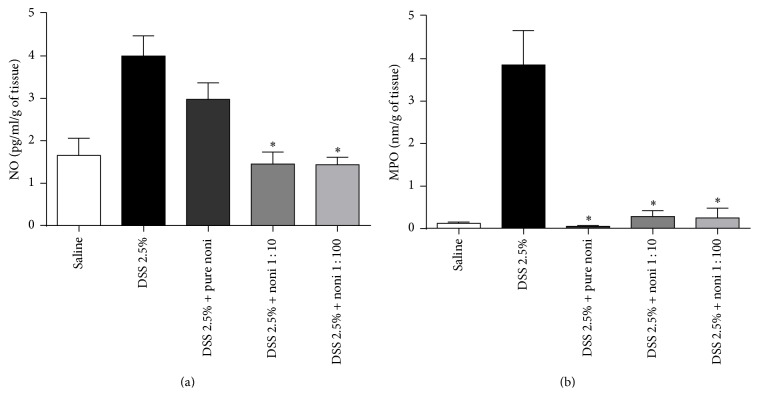
Noni fruit juice consumption inhibits inflammation by reducing nitric oxide and myeloperoxidase activities. C57BL/6 mice were exposed to dextran sulfate sodium (DSS) 2.5% and treated daily with noni fruit juice. On day 9, the mice were euthanized in order to obtain intestinal sections. Quantification of intestinal production of nitric oxide (a) and myeloperoxidase (MPO) activity, expressed as picogram per milliliter per gram of tissue (pg/ml/g) and as optic density per gram of tissue (nm/g of tissue), respectively. Data are represented as mean ± SEM. ^*∗*^*p* < 0.05.

The production of NO was reduced in the mice treated with noni fruit juice diluted 1 : 10 and 1 : 100 in relation to those that received pure noni or DSS (Figure 3(a)). Moreover, regardless of the concentration used, mice treated with noni fruit juice had reduced activity of MPO (Figure 3(b)) in comparison with DSS-exposed mice. Even though weight loss and disease outcome were not affected by noni fruit consumption, the improved intestinal architecture and the reduced activity of enzymes responsible for inflammation were associated with fruit juice consumption in a dose-dependent manner.

### 3.3. Noni Fruit Juice Consumption Reduces Key Inflammatory Cytokines in the Intestine in a Dose-Dependent Manner

**Figure 4:**
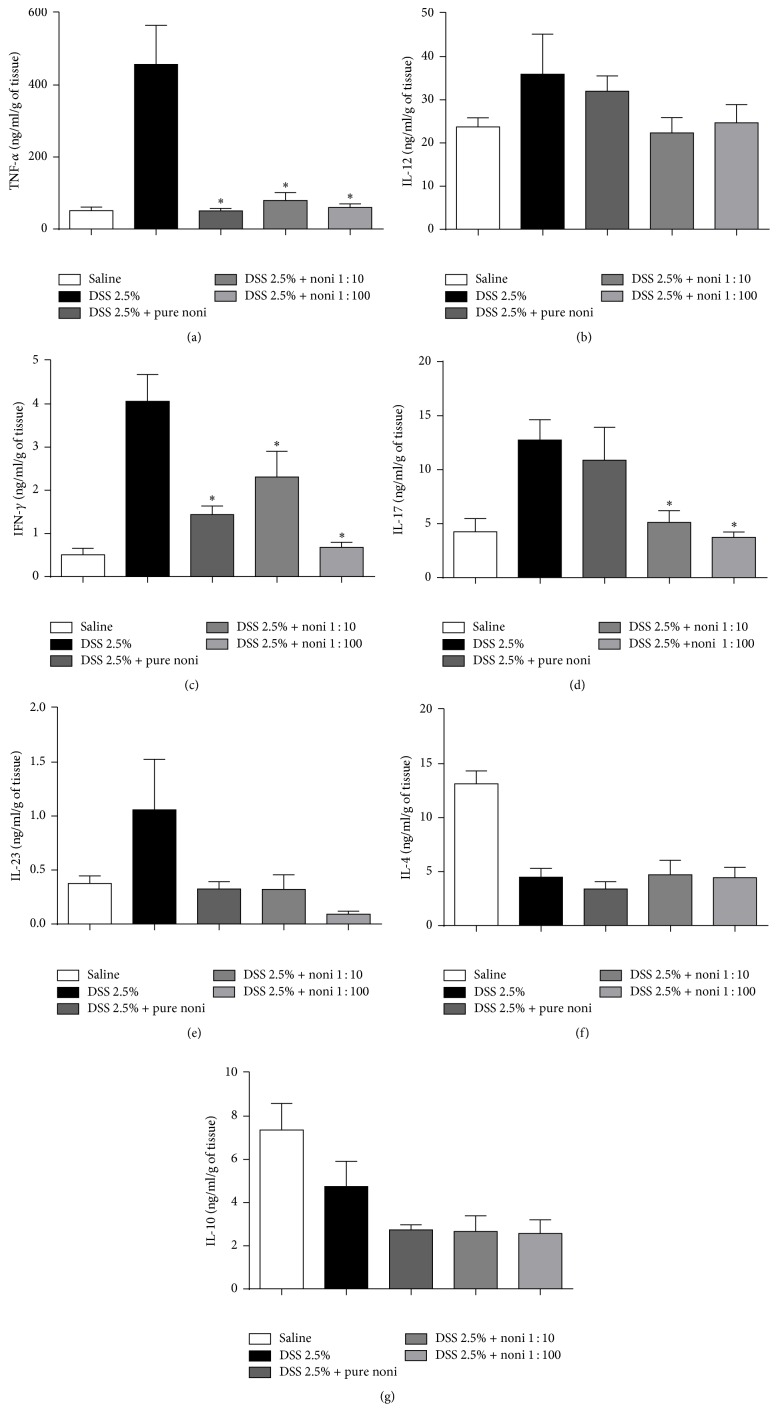
Noni fruit juice consumption reduces key inflammatory cytokines in the intestine in a dose-dependent manner. C57BL/6 mice were exposed to dextran sulfate sodium (DSS) 2.5% and treated daily with noni fruit juice. Enzyme-linked immunosorbent assay (ELISA) was performed in gut homogenates. (a) TNF-*α*, (b) IL-12, (c) IFN-*γ*, (d) IL-17, (e) IL-23, (f) IL-4, and (g) IL-10. Results were expressed as nanograms of cytokine per milliliter per gram of tissue (ng/ml/g of tissue), normalized by tissue dry weight. Data are represented as mean ± SEM. ^*∗*^*p* < 0.05.

Finally, we aimed to evaluate whether the improvement in intestinal architecture could also be associated with the modulation of key cytokines associated with disease worsening/protection. Mice treated with noni fruit juice, regardless of the concentration used, showed reduced production of inflammatory cytokines TNF-*α* and IFN-*γ* (Figures 4(a) and 4(c), resp.). A reduction in IL-17, another key cytokine associated with disease worsening, was observed only in mice treated with 1 : 10 and 1 : 100 dilutions (Figure 4(e)). Nevertheless, there were no differences in the production of IL-12 (Figure 4(b)), IL-4 (Figure 4(d)), IL-23 (Figure 4(f)), and IL-10 (Figure 4(g)). Taken together, these results suggest that improved intestinal architecture might also be associated with the local reduction of key inflammatory cytokines.

## 4. Discussion

The results presented herein demonstrate that noni fruit juice can reduce key inflammatory cytokines involved in the development of intestinal inflammation. Furthermore, treatment using fruit juice was also shown to be able to improve intestinal architecture, mainly when the dilution 1 : 10 was used. However, at least apparently, no effects were detected on the presentation of clinical signs of disease.

The beneficial properties of treatment using noni fruit juice in colitis control were partially attributed to an improvement in intestinal architecture along with a reduction in inflammatory infiltrate. This scenario was followed by a decrease in the activity of NO (only when noni fruit juice was diluted 1 : 10 and 1 : 100) and MPO (at any concentration). NO is a strong proinflammatory mediator mainly derived from inducible nitric oxide synthase (iNOS) after stimulation with bacterial endotoxins and inflammatory cytokines such as IL-1*β*, TNF-*α*, and IFN-*γ*, in different cell types, including macrophages, neutrophils, endothelial cells, and smooth muscle cells [21, 22]. Overexpression of iNOS, especially at mucosal sites, such as gastrointestinal tract, is reported to be associated with the development of inflammatory diseases, including IBD [23]. In this context, the production of high NO levels by infiltrating cells, such as macrophages, neutrophils, and lymphocytes, as well as by colon epithelial cells, was described to be directly associated with local tissue damage and disease worsening in IBD [24]. This observation was reinforced by the fact that the overexpression of iNOS induced by the inflammatory cytokines IL-1*β*, TNF-*α*, IFN-*γ*, IL-6, IL-17, and IL-23 was found in the plasma, in lamina propria mononuclear cells, and in colon epithelial cells of IBD patients and mice with intestinal inflammation [25–28], thus reinforcing the role of iNOS derivatives such as NO, and inflammatory cytokines, in the disease worsening and outcome. Therefore, it seems reasonable to believe that therapies aiming to modulate such aspects may represent an important tool to constrain inflammation and disease progression. In this context, monotropein isolated from the roots of* Morinda officinalis*, a member of the family Rubiaceae like* M. citrifolia*, reduced the in vitro production of NO in murine macrophages after stimulation with LPS in a dose-dependent manner [29]. A positive correlation between NO and the severity of IBD was proposed in clinical studies describing the occurrence of high levels of nitrite/nitrate in the plasma, urine, and lumen of IBD patients [27, 30, 31]. Shin et al. also demonstrated the activity of monotropein when mice were exposed to 4% DSS for 9 consecutive days. In this case, the treatment was able to reduce the activity of the inflammatory players COX-2 and MPO [29]. The study of MPO activity is a critical marker of neutrophil infiltration in the intestinal mucosa [32]. Indeed, studies using different protocols to induce colitis have demonstrated a positive correlation between the determination of MPO activity and disease severity [33–36]. Although the expressions of COX-2 and iNOS were not determined in our study, these results suggest that different members of Rubiaceae family can modulate inflammation in a similar way, thus inhibiting the progression and severity of DSS-induced colitis, using analogous mechanisms. The capability to modulate key inflammatory components by therapies aiming to control the activity of different cell types, such as macrophages, besides reducing the production of cytokines and NO, such as that observed in our study when NFJ was used, represents a promising approach to constrain the progression of inflammation in IBD. The control of inflammation by these mechanisms may explain the maintenance of intestinal architecture observed in our study when the dilutions 1 : 10 and 1 : 100 were used.

The complex and heterogeneous mechanisms associated with the development of intestinal inflammation include host genetics, environmental triggers, and disorders both in microbiota composition and in immune balance [7]. The severity of bowel inflammation is associated with increased production of proinflammatory cytokines (e.g., TNF-*α*, IFN-*γ*, IL-6, IL-17, and IL-23), which makes therapies aiming to reduce the levels of these cytokines relevant. In our study, treatment with noni fruit juice, in a dose-dependent manner, was able to reduce the production of TNF-*α*, IFN-*γ*, and IL-17 in the intestine.

TNF-*α* is one of the central players in the development of intestinal inflammation [37], and it is increased in the intestinal mucosa of patients with IBD [38]. This cytokine also has a pivotal role in the production of NO, and it increases the production of metalloproteinase, which contributes to the loss of epithelial integrity [39] and to disease worsening. The effects of TNF-*α* are mediated by two receptors, TNF receptor-1 (TNFR-1) and TNF receptor-2 (TNFR-2). The former can be expressed in either immune or nonimmune cells, resulting in the activation of NF-*κ*B, cytotoxicity, and production of inflammatory cytokines [40]. In our study, treatment with noni fruit juice considerably reduced the production of TNF-*α* in the intestine, regardless of the dilution used. This modulation could be, at least, partly attributed to the inhibition of NF-*κ*B by ascorbic acid and flavonoid glycoside, which were isolated from fermented noni fruit juice [41]. However, it is possible to speculate that these molecules might reduce the levels of TNF-*α* in the intestine by downregulating TNFR-1. Indeed, due to the importance of this cytokine in the development and aggravation of IBD, therapies aiming at targeting TNF-*α* to prevent the development of intestinal inflammation could be useful. The administration of monoclonal antibodies against IL-6 and TNF-*α* was able to reduce disease severity and attenuate intestinal inflammation in DSS-induced colitis [42]. Nevertheless, a substantial number of patients lose responsiveness especially due to production of antidrug antibodies and accelerated drug clearance [43], and that reinforces the need for new therapeutic approaches aiming to better control IBD progression and to reduce side effects.

IFN-*γ* is a proinflammatory cytokine produced by a broad range of cells, including T helper cells (CD4^+^T cells) and cytotoxic T cells (CD8^+^T cells), natural killer cells, and group 1 innate lymphoid cells [44]. A higher frequency of CD4^+^T cells and CD8^+^T cells producing IFN-*γ* was shown in patients with IBD in comparison with their control counterparts [44]. Both IFN-*γ* and TNF-*α* are increased in the mucosa of IBD patients and act synergistically contributing to the development and maintenance of inflammation that culminates in barrier breakdown [45]. These cytokines were shown to disrupt intercellular junction proteins under inflammatory conditions, which are not observed in noninflamed areas of unhealthy tissue [46]. Some of the mechanisms underlying these disorders include epithelial apoptosis [47] and reduced transcription of tight-junction proteins [48]. Therefore, therapies aiming to reduce this cytokine might be helpful in controlling inflammation and improving disease outcome. In this context, a short-term glucocorticoid treatment of mice exposed to DSS for six days was able to reduce the frequency of IFN-*γ*-producing CD4^+^ cells in the spleen and to decrease the expression of this cytokine and IL-1*β* in the intestine [49]. This reduction in the activity of IFN-*γ* was followed by an improvement in the clinical outcome and restoration of immune balance [49]. Additionally, the beneficial effects associated with the reduction of inflammatory cytokines such as IL-1*β* and IFN-*γ* were further elucidated in a murine model of colitis. In this case, colitis was induced by intracolonic administration of dinitrobenzene sulfonic acid (DNBS), and mice were treated using a nonpsychotropic cannabinoid, known as cannabigerol [50]. Even though the effects of noni fruit juice treatment on apoptosis and intestinal permeability were not investigated in this study, the improvement in intestinal architecture and the reduction in inflammatory infiltrate could be partly attributed to the reduction of IFN-*γ*.

IL-17-producing cells are involved in the pathogenesis of numerous inflammatory and autoimmune diseases, and they have been shown to mediate disease pathogenesis in IBD [51]. The role of Th17 cells in the pathogenesis of IBD was primarily attributed to a mutation in the* IL-23R* gene, which was identified in IBD patients and which regulates the production of Th17-related cytokines [52]. Indeed, an increased production of IL-17 by lamina propria cells in both UC and CD had already been shown [53]. Furthermore, cytokines produced by Th17 cells, such as IL-17 and IL-21, were found to upregulate the expression of TNF-*α*, IL-1*β*, IL-6, and IL-8 and the recruitment of neutrophils [54], which may contribute to IBD worsening. In addition to the classic role of Th17 lymphocytes in IBD pathogenesis, group 3 innate lymphoid cells have been also implicated in IL-17 production and disease pathogenesis in both experimental models and human subjects [55, 56]. In our study, reduced levels of IL-17 were detected in the colon of mice treated with noni fruit juice, but only when the 1 : 10 and 1 : 100 dilutions were used. Although we did not identify whether the reduction in IL-17 in the intestine was more associated with innate or adaptive immunity, or both, we cannot underestimate the importance of therapies aiming to produce these cytokines in order to inhibit inflammation in colitis.

Overall, our data showed that the treatment with noni fruit juice plays an important role in inhibiting inflammation during the development of experimental colitis. One of the key aspects regarding the use of fruit juice as a therapeutic option concerns its immune modulatory effect, which has a consequent impact on the improvement of intestinal architecture. Nonetheless, further studies must be performed in order to elucidate the molecules underlying the reduction of inflammatory cytokines in this model.

## Figures and Tables

**Table 1:** Histopathological scores of mice exposed to DSS in different conditions.

	DSS 2.5%	DSS 2.5% + pure noni	DSS 2.5% + noni 1 : 10	DSS 2.5% + noni 1 : 100
Lamina propria edema	Moderate	Moderate	Mild	Moderate
Submucosal edema	Severe	Moderate	Moderate	Moderate
Mucosal mononuclear infiltrate	Moderate	Mild	Mild	Moderate
Submucosal mononuclear infiltrate	Moderate	Mild	Mild	Mild
Intestinal crypts	Absent	Continuous	Irregular	Irregular
